# Posterior arm delivery versus the shoulder shrug maneuver in shoulder dystocia management: A simulation‐based comparative study

**DOI:** 10.1002/ijgo.70375

**Published:** 2025-07-17

**Authors:** Marcos Javier Cuerva, Celia Sanchez‐Giron, Mariona Cruset, Richard Bryan Hallam, Carolina Fernandez‐Cuesta, Ana Llamazares, Francisco Lopez, Marta Cortes, José Luis Bartha

**Affiliations:** ^1^ Department of Obstetrics Hospital Universitario La Paz Madrid Spain; ^2^ School of Medicine Universidad Autónoma de Madrid Madrid Spain; ^3^ School of Medicine Universidad Alfonso X el Sabio Madrid Spain; ^4^ Independent Biotechnology Consultant Madrid Spain; ^5^ Independent Design/Engineering Consultant, Freelance Madrid Spain

**Keywords:** emergency obstetrics, obstetric simulation, posterior arm delivery, shoulder dystocia, shoulder shrug maneuver

## Abstract

**Objective:**

To compare posterior arm delivery and the shoulder shrug maneuver in shoulder dystocia.

**Methods:**

A prospective, randomized, simulation‐based experimental study was conducted with six physicians managing 96 simulated shoulder dystocia cases. Participants included three obstetricians with more than 15 years of experience and three trainees. Variables collected included perineal pressure, maneuver time, simulated humeral fractures, first‐attempt success, and perceived difficulty.

**Results:**

Posterior arm delivery exerted significantly less maximum perineal pressure than the shoulder shrug maneuver (median 12.5 mm Hg, interquartile range (IQR) 7.0–22.5 mm Hg versus median 22.0, IQR 13.0–37.0 mm Hg; *P* = 0.002). Obstetricians with greater experience applied less perineal pressure and performed maneuvers faster compared with trainees. Two simulated humeral fractures occurred, one with each maneuver, both involving trainees. No significant differences were observed regarding hand dominance or hand size.

**Conclusion:**

Posterior arm delivery showed lower perineal pressure compared with the shoulder shrug maneuver, suggesting reduced maternal and neonatal risk. Clinical experience positively influenced outcomes, supporting recommendations for experienced obstetricians to lead shoulder dystocia management. Posterior arm delivery is recommended as the first‐choice internal maneuver when addressing the posterior shoulder in shoulder dystocia cases.

## 1 INTRODUCTION

Shoulder dystocia is an unpredictable and potentially severe obstetric emergency. Reported incidence rates of shoulder dystocia vary widely in the literature, largely influenced by the diagnostic criteria employed, ranging from 0.5% to more than 10%, with a generally accepted estimate between 2% and 3% of all births.^1^, ^2^ The primary complication associated with shoulder dystocia is brachial plexus injury, occurring in 0.5–3 per 1000 births.^3^, ^4^, ^5^ It is important to highlight that shoulder dystocia is the principal risk factor for this complication.

No single maneuver has been demonstrated to be clearly superior to the others in the management of shoulder dystocia. Each maneuver carries distinct risks of neonatal injury. Although it has traditionally been considered appropriate to begin with first‐line maneuvers and proceed to second‐line techniques in case of failure, there is currently consensus that no universal sequence applies to all cases.^6^, ^7^

Posterior arm delivery, also known as manual extraction of the posterior arm or Jacquemier‐Barnum maneuver, has been reported to have the highest success rate, approximately 86%, although it carries a risk of complications such as humeral fracture.^6^ Other techniques targeting the posterior shoulder have also been described, with similar success rates, such as the shoulder shrug maneuver.^6^, ^8^

The quality and extent of scientific evidence supporting these maneuvers vary considerably. The posterior arm delivery is supported by substantial clinical experience and numerous reports, but evidence for the shoulder shrug maneuver remains limited.^6^, ^9^ In order to refine the technique and enable comparison between the different maneuvers, simulation‐based training represents a safe and effective approach, allowing for a high number of repetitions without any risk to patients.^10^, ^11^

The aim of this study was to compare the performance of posterior arm delivery and the shoulder shrug maneuver in a simulation model. The primary objectives were to evaluate the maximum pressure exerted on the perineum, the potential for neonatal injury, and the perceived difficulty associated with each maneuver. Secondary objectives included assessing whether the results were consistent according to the physician's level of experience and the use of the dominant versus non‐dominant hand.

## 2 MATERIALS AND METHODS

This was a simulation‐based, experimental, prospective, non‐blinded, randomized study. The Ethics Committee of Hospital Universitario La Paz granted an exemption from approval in November 2024, as no human participants were involved. Consequently, informed consent was not required. The institutional board approved the investigation protocol (PI‐6414).

This experimental study involved six physicians managing a total of 96 cases of shoulder dystocia using a simulation model. Of the six physicians, three were obstetricians with more than 15 years of experience, and three were medical residents.

**FIGURE 1:**
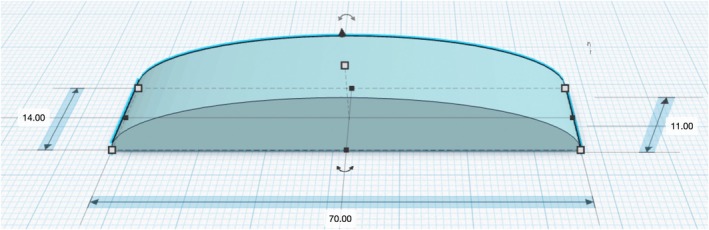
Custom‐designed piece. The geometry corresponds to a semi‐cylindrical segment. The piece measures 70 mm in width, with a maximum height of 11 mm and a depth of 14 mm.

**FIGURE 2:**
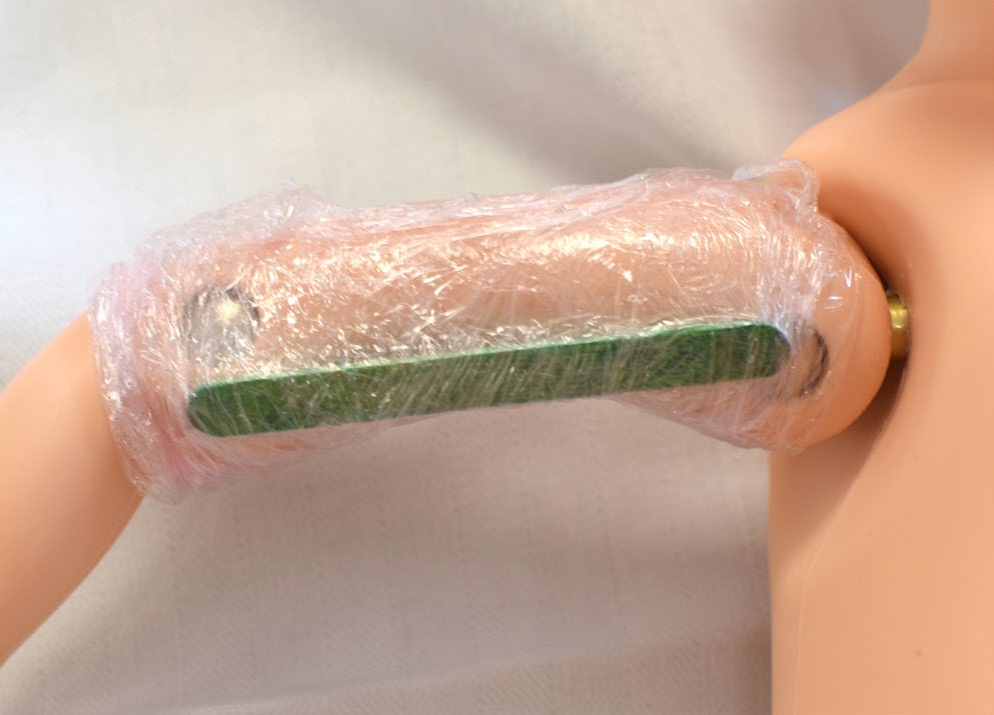
Wooden craft stick (73 × 7 × 2 mm) placed along the upper limb of the neonatal mannequin and secured with transparent plastic film. The stick extends from the metallic shoulder joint to the lateral epicondyle, following the humeral axis.

The simulation was conducted with a PROMPT Flex—Birthing Simulator (Limbs & Things Ltd., Bristol, UK). Additionally, a piece was designed using the Tinkercad platform, which was subsequently printed with a 3D printer using TPE‐83A filament (Figure 1). This piece was then attached to the pubis to simulate a severe shoulder dystocia accurately. A LIM‐80175 Foam Support (Limbs & Things Ltd) was used to ensure that the placement of the neonate was identical in each simulation, eliminating the need for an assistant to hold the mannequin. A Koala pressure catheter (Laborie Medical Technologies Corp., Portsmouth, NH, USA) was placed in the perineal region of the simulator, positioned between the vagina and the anus. This design has been successfully used in a previous study on forceps.^12^ Finally, wooden craft sticks measuring 73 × 7 × 2 mm (Innspiro, Barcelona, Spain) were placed in both arms to assess potential humeral fractures (Figure 2). The complete design was selected after multiple material tests and consensus among three obstetricians with more than 20 years of experience, who confirmed its realism.

In the study, the maneuver to be performed and the fetal back position were randomized for each simulation. Independent simulation sessions were conducted for each participating physician. The maneuvers used were the posterior arm delivery (traditional posterior arm extraction or Jacquemier‐Barnum maneuver) and the shoulder shrug maneuver.

The posterior arm delivery involved reaching the posterior arm, reducing the biacromial diameter, and rotating the shoulders into the oblique pelvic dimension by pulling the posterior arm outward and upward (Video S1). The shoulder shrug maneuver used finger traction under the posterior axilla to rotate the shoulders and facilitate delivery (Video S2). Each maneuver was performed with the hand on the same side as the fetal back, that is, the hand “facing the baby”. If the fetal back was on the maternal left side, the left hand was used; if it was on the maternal right side, the right hand was used.

The following variables were collected from each simulation: use of the operator's dominant hand on the posterior arm, operator's experience (divided between trainees and obstetricians with >15 years of experience), surgical glove size, maneuver time, maximum perineal pressure, humeral injuries (indicated by damage to the wooden craft sticks), failure of the maneuver on the first attempt, and perceived difficulty according to a five‐point Likert scale.

### 2.1 Sample size calculation

The sample size calculation (number of simulations) was performed using data from a pilot study, in which eight cases resolved with posterior arm delivery showed a mean maximum perineal pressure of 17.1 mm Hg and a standard deviation of ±5.6 (these cases were only used to calculate the sample size and were not included in the final results of the study).

Considering an α level of 0.05, and a power (1 − *β*) of 80%, a sample size of 20 simulations in group 1 and 20 in group 2 were needed to detect a difference of 5 mm Hg. To allow independent comparisons between the use of the dominant hand and the non‐dominant hand, it was decided to perform 20 simulations with each maneuver in cases with the right fetal back position and an equal number in the left fetal back position.

Finally, to study the analysis of secondary variables, we decided to perform 24 simulations per maneuver and fetal back position, resulting in a total of 96 simulated shoulder dystocia cases. Therefore, each participating physician was required to manage a total of 16 shoulder dystocia cases.

### 2.2 Statistical analysis

The distribution of variables was evaluated through the Shapiro–Wilk test and by visual inspection of histograms. Continuous variables were summarized as medians with interquartile ranges (IQR), whereas categorical variables were presented as absolute and relative frequencies. Comparisons between groups were performed using the Mann–Whitney *U* test or the Kruskal‐Wallis H test for continuous data and either the two‐tailed *χ* ^2^ test or two‐tailed Fisher exact test for categorical data, as appropriate. To examine the association between independent variables and outcomes, a multiple linear regression analysis was conducted. Variables with a *P* value less than 0.25 in the univariate analysis were included in the multivariate model. A two‐sided *P* value less than 0.05 was considered statistically significant. The Bonferroni correction was applied to comparisons involving more than two groups to control for the increased risk of Type I error associated with multiple testing. All statistical analyses were carried out using SPSS software, version 22.0 (IBM, Armonk, NY, USA).

## 3 RESULTS

A total of 96 simulated shoulder dystocia cases were conducted as part of the study. These were equally divided between the two maneuvers being evaluated: 48 (50%) simulations were resolved using the posterior arm delivery technique and 48 (50%) were managed using the shoulder shrug maneuver. Half of the simulations were performed using the participants' dominant hand, and the other half with the non‐dominant hand.

Among the six participating physicians, three were obstetricians with more than 15 years of experience, and three were trainees. The group was composed of four female and two male participants. In terms of hand size, based on surgical glove sizes, one physician wore size 7.5, one wore size 7, two wore size 6.5, and two wore size 6.

Across all simulations performed in the study, the median maximum pressure exerted on the perineum was 16.5 (IQR 10.0–27.0) mm Hg, the maneuver time was 7.8 (IQR 6.0–10.0) seconds, and the perceived difficulty was rated at 2.5 (IQR 2.0–3.0). The wooden craft stick broke in two simulations (2.1%). Additionally, in one case (1.0%), the maneuver was unsuccessful on the first attempt and required a second attempt to resolve the dystocia.

A statistically significant difference was identified when comparing the two maneuvers in terms of perineal pressure. The shoulder shrug maneuver exerted significantly greater perineal pressure than the posterior arm delivery (22.0 (IQR 13.0–37.0) mm Hg versus 12.5 (IQR 7.0–22.5) mm Hg, *P* = 0.002) (Table 1).

**TABLE 1:** Comparison between different maneuvers, operator experience, use of the dominant hand, and surgical glove sizes 6 and 6.5 versus 7 and 7.5.^a^

	Jacquemier (*n* = 48)	Shoulder shrug (*n* = 48)	*P* value
Perineal pressure, mm Hg	12.5 (7.0–22.5)	22.0 (13.0–37.0)	0.002
Time, s	8.1 (6.0–10.5)	7.0 (5.6–9.0)	0.149
Difficulty	3.0 (2.0–3.0)	2.0 (2.0–3.0)	0.944
Humeral injury	1 (2.1%)	1 (2.1%)	>0.999

	>15 years of experience (*n* = 48)	Trainee (*n* = 48)	*P* value
Perineal pressure, mm Hg	13.5 (7.0–24.0)	20.5 (12.5–34.0)	0.015
Time, s	7.0 (5.1–8.6)	8.9 (6.7–11.0)	<0.001
Difficulty	2.0 (2.0–3.0)	3.0 (2.0–3.5)	0.118
Humeral injury	0 (0%)	2 (4.2%)	0.495

	Dominant hand (*n* = 48)	Non dominant hand (*n* = 48)	*P* value
Perineal pressure, mm Hg	15.5 (9.5–24.0)	18.0 (10.0–35.5)	0.245
Time, s	7.2 (5.7–10.0)	8.0 (6.0–9.8)	0.560
Difficulty	2.0 (2.0–3.0)	3.0 (2.0–3.0)	0.289
Humeral injury	2 (4.2%)	0 (0%)	0.495

	Glove sizes 7 and 7.5 (*n* = 24)	Sizes 6 and 6.5 (*n* = 72)	*P* value
Perineal pressure, mm Hg	13.5 (9.5–24.0)	18.5 (10.0–30.5)	0.290
Time, s	8.0 (5.8–8.9)	7.5 (6.0–10.0)	0.526
Difficulty	2.5 (2.0–3.0)	2.5 (2.0–3.0)	0.711
Humeral injury	0 (0%)	2 (4.2%)	0.495

^a^ Data are presented as medians (interquartile range), and absolute and relative frequencies for qualitative variables.

Operator experience was also associated with significant differences. Physicians with more than 15 years of experience exerted lower perineal pressure than trainees (13.5 (IQR 7.0–24.0) mm Hg vs. 20.5 (IQR 12.5–34.0) mm Hg, *P* = 0.015) (Table 1). Similarly, experienced physicians completed the maneuvers more quickly compared with trainees (7.0 (IQR 5.1–8.6) s vs. 8.9 (IQR 6.7–11.0) s, *P* < 0.001) (Table 1).

No significant differences were identified in the studied parameters between simulations performed with the dominant hand and those performed with the non‐dominant hand. Similarly, no differences were observed between male and female participants (the male participants wore surgical glove sizes 7 and 7.5) (Table 1). Additionally, no significant differences were found between the different glove sizes after adjusting the *α* value with the Bonferroni correction for multiple comparisons (Table S1).

The two cases in which the simulation indicated a potential humeral fracture involved trainees: one case using the posterior arm delivery maneuver and the other using the shoulder shrug maneuver. In both cases, the surgical glove sizes were 6 and 6.5, and both simulations were performed with the dominant hand.

The case in which a second attempt was necessary occurred during a simulation involving the shoulder shrug maneuver. This case was performed by a trainee, using her non‐dominant hand, and wearing a size 6.5 glove.

Finally, a multiple linear regression analysis was conducted to control for possible confounding factors, including the type of maneuver, the participant's level of experience, and the use of the dominant hand. The analysis showed that perineal pressure was independently associated with both the maneuver employed and the participant's level of experience (Table 2). In contrast, after adjusting for the type of maneuver and participant experience, procedure time was found to be independently associated solely with participant experience (Table 2).

**TABLE 2:** Multiple linear regression of variables predicting the pressure on the perineum and the duration of the maneuver.^a^

Variable	*B*	SE	*Β*	*t*	95% CI for *B*	*P*
Variables predicting the pressure on the perineum						
Maneuver	12.00	3.96	0.29	3.03	4.14–19.86	0.003
Experience	−10.75	3.96	−0.26	−2.71	18.61 to −2.90	0.008
Dominant hand	−7.75	3.96	−0.19	−1.96	−15.61 to 0.11	0.053
Constant	22.06	8.85	–	2.50	4.50–39.63	0.014
Variables predicting the duration of the maneuver (time)						
Maneuver	−0.32	1.15	−0.03	−0.28	−2.60 to 1.96	0.782
Experience	−2.82	1.15	−0.25	−2.46	−5.10 to −0.54	0.016
Constant	10.65	1.90	–	5.60	6.88–14.42	<0.001

Abbreviations: CI, confidence interval; SE, standard error.

^a^ Instrument: Maneuver—Shoulder shrug maneuver (Maneuver = 1) or Jacquemier (Maneuver = 0). Experience—>15 years (Experience = 1) or trainees (Experience = 0). Dominant hand: Dominant (Dominant hand = 1) or Non‐dominant (Dominant hand = 0).

## 4 DISCUSSION

Our study shows that the posterior arm delivery exerts significantly less perineal pressure compared with the shoulder shrug maneuver. This finding can be explained by the lack of necessity for traction and because the hand rapidly moves away from the posterior perineum towards the lateral area. This clinical finding is particularly significant because internal maneuvers involving manipulation of the posterior shoulder, although highly effective, inherently carry risks. Hence, prioritizing maneuvers associated with lower maternal and neonatal morbidity is crucial.

Both posterior arm delivery and the shoulder shrug maneuver achieve success rates exceeding 80%.^6^, ^8^ Consequently, maneuver selection should consider additional safety factors beyond success rates alone. It is known that increased perineal pressure is associated with higher neonatal and maternal morbidity.^13^ Additionally, the posterior arm delivery has more robust evidence, supported by reviews and meta‐analyses on the topic.^6^, ^9^

Shoulder dystocia is a known risk factor for severe perineal trauma, including obstetric anal sphincter injuries (OASI).^14^ Cases resolved with external maneuvers are associated with lower rates of maternal injury compared with those requiring internal manipulations.^15^ Posterior arm delivery has been associated with approximately a two‐fold increase in the likelihood of OASI, whereas other internal rotation maneuvers, such as the Woods screw and reverse Woods screw, have demonstrated even higher risks.^16^ In our study, perineal pressure was measured at the area between the vaginal and anal openings of the simulator, corresponding anatomically to the region at greatest risk for OASI. In this context, the shoulder shrug maneuver resulted in higher perineal pressure values compared with posterior arm delivery.

Regarding neonatal injuries, cerebral hypoxia is the most significant outcome following shoulder dystocia. It is closely associated with the interval between shoulder impaction and birth. A prolonged head‐to‐body delivery interval increases the risk of hypoxic–ischemic encephalopathy and severe neonatal acidosis.^17^, ^18^ In our study, no statistically significant differences were observed in the time required to perform the shoulder shrug maneuver compared with posterior arm delivery, suggesting that, in terms of timing, both techniques are equally effective.

A specific complication associated with internal maneuvers involving manipulation of the posterior arm is humeral fracture. Current literature estimates the incidence of this complication to range between approximately 2% and 7%.^19^, ^20^, ^21^ Considering that forces in the range of 10 to 20 Newtons are estimated to be sufficient to fracture an infant humerus, our study aimed to evaluate this specific risk using wooden craft sticks.^22^ However, the enhanced realism of our simulation led to the detection of only two simulated humeral fractures, one with each maneuver. Consequently, no conclusions regarding differences between the two techniques could be drawn. Notably, both fractures occurred among trainees; no such incidents were reported among experienced obstetricians participating in the study.

As in other areas of obstetrics, the experience level of the obstetrician is an essential factor that positively influences clinical outcomes. Multiple clinical guidelines addressing shoulder dystocia explicitly recommend that the most experienced obstetrician available should take leadership during management of this obstetric emergency to optimize maternal and neonatal safety.^20^, ^21^, ^23^, ^24^ Consistent with this recommendation, our study demonstrated that greater clinician experience was independently associated not only with reduced perineal pressure but also with more rapid and efficient execution of maneuvers.

Another aspect evaluated in our study was the potential influence of hand dominance and hand size on the performance of posterior arm delivery and the shoulder shrug maneuver. Contrary to expectations, no significant differences were found in perineal pressure, perceived difficulty, or execution time when comparing the use of the dominant versus the non‐dominant hand for either maneuver. Similarly, hand size did not demonstrate any correlation with the variables assessed, although it is important to note that the largest glove size among participants was 7.5. Regarding the use of the non‐dominant hand, we believe that our findings may be explained by the fact that obstetric training emphasizes the development of proficiency with both hands, with ambidexterity being considered a highly valuable skill in clinical practice and professional training.^25^ Regarding hand size, it is possible that practitioners with smaller hands may adopt different techniques or hand positions, potentially altering their performance, which could explain why perineal pressure remains similar despite the smaller hand volume.^26^

In terms of strengths, this study represents the first direct comparison between the two most widely used maneuvers involving the posterior arm in the management of shoulder dystocia: the posterior arm delivery and the shoulder shrug maneuver. Furthermore, the use of a high‐fidelity simulation model, combined with a standardized support system for the manikin, allowed for the reproduction of shoulder dystocia cases with consistent severity across all scenarios. This methodologic rigor enhances the reliability, reproducibility, and internal validity of our findings, minimizing variability attributable to external factors.

On the other hand, some limitations should be acknowledged. One of the primary limitations of simulation‐based research is its inability to replicate the emotional and psychological stress experienced by clinicians during real‐life obstetric emergencies. This lack of emotional stress may have influenced participants' perceptions, potentially explaining the absence of differences in perceived difficulty between maneuvers or among participants with varying levels of clinical experience. In actual clinical practice, factors such as time pressure, anxiety, and the potential consequences of adverse outcomes can significantly impact performance.^27^ Another limitation lies in the fact that the materials used in the simulations differ from biologic tissues. For example, while wooden craft sticks may require a similar amount of force to break as infant humeri, they do not replicate the distinct biomechanical properties of neonatal bone. Finally, the fact that the study was conducted at a single institution may limit the generalizability of the results. However, at our center, structured shoulder dystocia training sessions are held on a monthly basis and include both posterior arm delivery and the shoulder shrug maneuver. This supports our intention to evaluate the maneuvers as they are described in the literature, rather than based on local practice. Nonetheless, future multicenter studies will be necessary to confirm the findings and ensure high external validity.

In conclusion, our study shows advantages associated with the posterior arm delivery compared with the shoulder shrug maneuver. For this reason, we consider posterior arm delivery to be the first internal maneuver of choice when addressing the posterior arm in cases of shoulder dystocia. Furthermore, obstetricians with greater clinical experience achieved superior outcomes in our study, supporting the recommendation that the most experienced clinician available should lead the decision making process and perform the maneuvers whenever possible.

## AUTHOR CONTRIBUTIONS

MJC contributed to protocol/project development, data collection, and manuscript writing. CS contributed to protocol/project development, data analysis, and manuscript writing. MCr and RBH contributed to protocol/project development and manuscript editing. CF, AL, MCo, and FL contributed to data collection and manuscript writing/editing; and JLB contributed to protocol/project development, validation of data analysis, and manuscript reviewing and editing. All authors contributed to editorial changes in the manuscript and read and approved the final manuscript. All authors have participated sufficiently in the work and agreed to be accountable for all aspects of the work.

## CONFLICT OF INTEREST STATEMENT

The authors have no conflicts of interest.

## Supporting information


**TABLE S1.** Comparison between different surgical glove sizes.

**VIDEO S1.** Posterior arm delivery or Jacquemier‐Barnum maneuver. The abdominal skin was removed to allow visualization of internal maneuvers during the recordings.

**VIDEO S2.** Shoulder shrug maneuver. The abdominal skin was removed to allow visualization of internal maneuvers during the recordings.

## Data Availability

Data available on request from the authors.
