# Supplementary material for: Posterior arm delivery versus the shoulder shrug maneuver in shoulder dystocia management: A simulation‐based comparative study
Source: Int J Gynaecol Obstet. 2025 Jul 17;172(1):558–64. doi: 10.1002/ijgo.70375 (PMC12724062; doi:10.1002/ijgo.70375)
Supplement: Supplementary file 1 — TABLE S1. Comparison between different surgical glove sizes. [file IJGO-172-558-s001.docx]

**Supplementary table 1.** **Comparison between different surgical glove sizes.**

|  | **Glove size 6.0 (n=32)** | **Glove size 6.5 (n=32)** | **Glove size 7.0 (n=16)** | **Glove size 7.5 (n=16)** | ***P*** |
| --- | --- | --- | --- | --- | --- |
| Perineal pressure (mmHg) | 15.0 (8.0; 22.0) | 22.5 (12.5; 50.0) | 12.5 (8.0; 27.0) | 14.0 (10.0; 23.5) | 0.071 |
| Time (seconds) | 8.8 (5.1; 11.5) | 7.0 (6.4; 9.2) | 8.5 (7.5; 11.5) | 5.9 (5.3; 8.0) | 0.024 |
| Difficulty | 3.0 (2.0; 3.0) | 2.0 (2.0; 3.0) | 2.0 (2.0; 3.0) | 3.0 (2.0; 3.0) | 0.787 |
| Humeral injury | 1 (3.1%) | 1 (3.1%) | 0 (0.0%) | 0 (0.0%) | 0.796 |

Data are presented as medians (interquartile range), and absolute and relative frequencies for qualitative variables.

The adjusted level of significance after applying the Bonferroni correction is 0.0083.
